# Trachoma then and now: update on mapping and control

**Published:** 2018-02-08

**Authors:** Anthony W Solomon, Paul M Emerson, Serge Resnikoff

**Affiliations:** 1 Medical Officer for Trachoma: Department of Control of Neglected Tropical Diseases, World Health Organization, Geneva, Switzerland.; 2 Director: International Trachoma Initiative, Decatur GA, USA.; 3 Chair: International Coalition for Trachoma Control; President and Chair: Organisation pour la Prévention de la Cécité, Paris, France.

**In the last 30 years, there has been rapid progress towards ending the suffering and blindness caused by trachoma, with five countries being validated as having achieved elimination. However, many challenges remain.**

**Figure:**
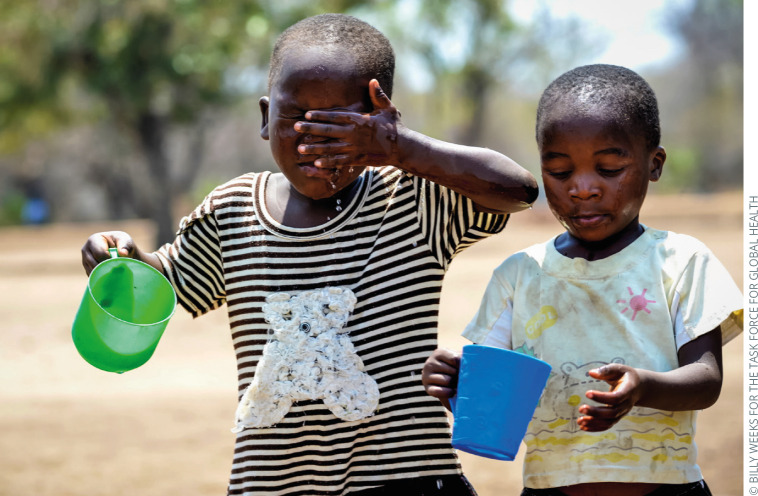
Facial cleanliness is an important part of the trachoma elimination strategy. MALAWI

When the first issue of the *Community Eye Health Journal* was being sent to readers around the world in 1988, trachoma was at a turning point. One of the two foundational clinical trials establishing the effectiveness of the bilamellar tarsal rotation procedure for trachomatous trichiasis had just been completed; the other was about to start.^1,2^ The pharmacokinetics and antimicrobial spectrum of azithromycin, a recently discovered macrolide antibiotic, were in the process of being defined.^3,4^ The epidemiological association between a lack of facial cleanliness and the presence of active trachoma was becoming clearly established^5,6^ and the World Health Organization's (WHO'S) simplified grading system had just been published,^7^ providing non-specialist health personnel working in endemic communities with a means to clearly and quickly identify and record the burden of disease. Additionally, the first national survey of the prevalence and causes of blindness in a country in Africa had just finished; it was conducted in The Gambia and suggested that 17% of all blindness there was due to trachoma.^8^

**Figure:**
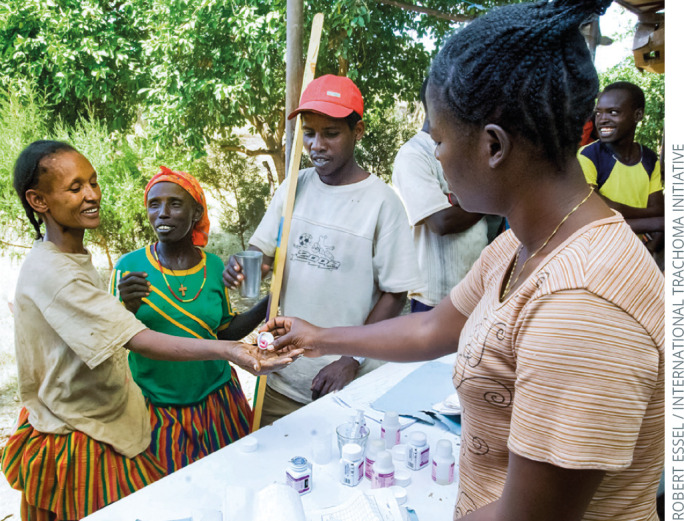
Mass distribution of antibiotics. ETHIOPIA

These developments led, in the subsequent decade, to:

- Demonstration of the effectiveness of single-dose oral azithromycin against active trachoma,^9^ successful trials of azithromycin mass drug administration,^10^ and the initiation of a donation scheme by Pfizer, Inc., the manufacturer of azithromycin^11^
- Landmark community randomised trials investigating intensive facial cleanliness campaigns and fly control for reducing the prevalence of active disease^12,13^
- The 1993 WHO endorsement of the “SAFE strategy” (surgery, antibiotics, facial cleanliness and environmental improvement) for trachoma elimination^14^
- Establishment, at the end of 1996, of the WHO Alliance for the Global Elimination of Trachoma by 2020(GET2020)^15^
- The 1998 World Health Assembly resolution 51.11, which called on endemic countries and WHO to take all actions necessary to achieve the GET2020 goal.^16^

## The current landscape

As a consequence of the above, the landscape for trachoma now looks very different. The SAFE strategy is being implemented, partially or at scale, in at least 31 countries. In 2016, the year for which the most recent global data are available, more than 260,000 people had their trachomatous trichiasis managed, while more than 85 million people received antibiotics for trachoma.^17^ Pfizer's azithromycin (Zithromax^®^) donation scheme has ramped up from one hundred thousand doses shipped in 1999, to more than one hundred and twenty million doses shipped in 2016.^18^ As a result, global antibiotic coverage is expected to increase again from 2016 to 2017.^17^

Much of these recent increases in output of the SAFE strategy's surgery and antibiotic components rely on data from the Global Trachoma Mapping Project (GTMP),^19^ which from 2012–2016 completed population-based prevalence surveys in 1546 districts of 29 countries, adding to the 1,115 districts for which data had previously been amassed.^20^ By working with health ministries to generate gold-standard data on trachoma prevalence within a culture of collaboration, openness and commitment to quality,^21,22^ the GTMP helped foster a spirit of genuine collaboration within the trachoma elimination community, shone a light that has helped the rest of the world to see the ongoing public health tragedy of trachoma, and provided the district-by-district justification required to initiate interventions.

Financial resources to complement the continuing azithromycin donation have followed, with new or renewed contributions from a committed group of bilateral agencies, private foundations, non-governmental development organisations, service organisations and individual donors.

Concrete proof of progress against disease is now available. The number of people worldwide who need operations for trichiasis is thought to have decreased from 8.2 million in 2007^23^ to 2.8 million in 2016. Similarly, the number of people worldwide living in districts where the A, F and E components of SAFE need to be implemented for trachoma elimination purposes is thought to have decreased from 1,244 million in 2007 to 190 million in 2016.^17,23^ Oman, Morocco, Mexico, Lao People's Democratic Republic and Cambodia have now all been officially validated as having eliminated trachoma as a public health problem, while a further six countries (China, Ghana, Iraq, Islamic Republic of Iran, Myanmar and The Gambia) have reported achieving elimination prevalence targets.^24^

The *Community Eye Health Journal* has been a part of this journey. In its first 100 issues, it has published more than 50 excellent articles about trachoma (**www.cehjournal.org/category/trachoma/**), providing a critical forum for education, information, debate and reflection. We congratulate the *Journal* on its century, and thank the editors, donors and readers who have contributed so much to international efforts against trachoma to date.

It would be wrong, however, to imply through these notes of congratulation that the race against trachoma has now been successfully run, or even that we could coast in from here to the finish line. Significant challenges remain.

1. There is an urgent need to address the remaining gap between the resources that have been committed and those that will be required.
2. Important work is also needed on a number of technical issues, including:
  - How best to manage post-operative trachomatous trichiasis
  - How to most efficiently deliver water, hygiene and sanitation interventions to cut transmission of ocular *Chlamydia trachomatis*
  - How to undertake post-validation surveillance of previously-endemic districts, in order to guard against recrudescence of infection and disease.

It is our hope that with the ongoing political support of endemic country governments, current programmatic momentum, the continuing commitment of our many partners, and the relevance of our work to a multitude of cross-cutting targets within the Sustainable Development Goals,^25^ the end of trachoma can be achieved.

**Figure 1:**
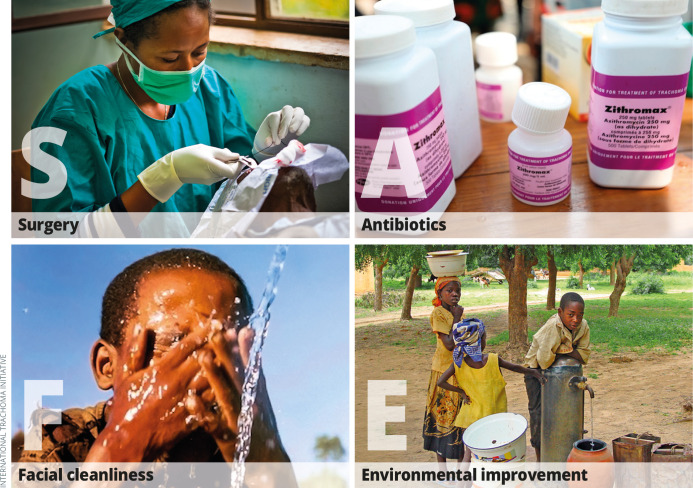
The SAFE strategy for trachoma control

